# Identification of research hypotheses and new knowledge from scientific literature

**DOI:** 10.1186/s12911-018-0639-1

**Published:** 2018-06-25

**Authors:** Matthew Shardlow, Riza Batista-Navarro, Paul Thompson, Raheel Nawaz, John McNaught, Sophia Ananiadou

**Affiliations:** National Centre for Text Mining, University of Manchester, Manchester, UK

**Keywords:** Text mining, Events, Meta-knowledge, Hypothesis, New knowledge

## Abstract

**Background:**

Text mining (TM) methods have been used extensively to extract relations and events from the literature. In addition, TM techniques have been used to extract various types or *dimensions* of interpretative information, known as Meta-Knowledge (MK), from the context of relations and events, e.g. negation, speculation, certainty and knowledge type. However, most existing methods have focussed on the extraction of individual dimensions of MK, without investigating how they can be combined to obtain even richer contextual information. In this paper, we describe a novel, supervised method to extract new MK dimensions that encode *Research Hypotheses* (an author’s intended knowledge gain) and *New Knowledge* (an author’s findings). The method incorporates various features, including a combination of simple MK dimensions.

**Methods:**

We identify previously explored dimensions and then use a random forest to combine these with linguistic features into a classification model. To facilitate evaluation of the model, we have enriched two existing corpora annotated with relations and events, i.e., a subset of the GENIA-MK corpus and the EU-ADR corpus, by adding attributes to encode whether each relation or event corresponds to Research Hypothesis or New Knowledge. In the GENIA-MK corpus, these new attributes complement simpler MK dimensions that had previously been annotated.

**Results:**

We show that our approach is able to assign different types of MK dimensions to relations and events with a high degree of accuracy. Firstly, our method is able to improve upon the previously reported state of the art performance for an existing dimension, i.e., Knowledge Type. Secondly, we also demonstrate high F1-score in predicting the new dimensions of Research Hypothesis (GENIA: 0.914, EU-ADR 0.802) and New Knowledge (GENIA: 0.829, EU-ADR 0.836).

**Conclusion:**

We have presented a novel approach for predicting New Knowledge and Research Hypothesis, which combines simple MK dimensions to achieve high F1-scores. The extraction of such information is valuable for a number of practical TM applications.

**Electronic supplementary material:**

The online version of this article (10.1186/s12911-018-0639-1) contains supplementary material, which is available to authorized users.

## Background

The goal of information extraction (IE) is to automatically distil and structure associations from unstructured text, with the aim of making it easier to locate information of interest in huge volumes of text. Within biomedical research articles, the textual context of a particular piece of knowledge often provides clues as to its current status along the ‘research journey’ timeline. Sentences (1)–(3) below exemplify a number of different points along the research timeline regarding the establishment of an association between *Interleukin-17 (IL-17)* and *psoriasis*. The association is firstly introduced in (1) as a hypothesis to be investigated. In (2), which is taken from the same paper [1], the putative association is backed up by initial experimental evidence. Sentence (3) comes from a paper published 10 years later [2], by which time the association is presented as widely accepted knowledge, presumably on the basis of many further positive experimental results.

(1) *‘To investigate the role of Interleukin-17 (IL-17) in the pathogenesis of psoriasis...’*

(2) *‘These findings indicate that up-regulated expression of IL-17 might be involved in the pathogenesis of psoriasis.’*

(3) *‘IL-17 is a critical factor in the pathogenesis of psoriasis and other inflammatory diseases.’*

There is a strong need to identify different types of emerging knowledge, such as those shown in sentences (1–2), in a number of different scenarios. It has been shown elsewhere that incorporating this type of information improves the automated curation of biomedical networks and models [3].

**Fig. 1:**
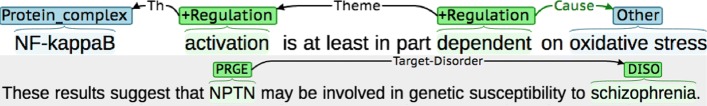
An example of two sentences, one containing events and the other containing one relation. The first sentence shows two events. The first event in the sentence concerns the term ‘activation’ which is a type of positive regulation. The theme of this event is ‘NF-kappaB’, indicating that this protein is being activated. The next event in the sentence is centered around ‘dependent’ which is a type of positive regulation. This event has the cause ‘oxidative stress’ and its theme is the first event in the sentence. The example of a relation between two entities is, in contrast to the event, clearly much more simple. The relation indicates that NPTN is related to Schizophrenia in a relation that can be categorised as ‘Target-Disorder’

In processing sentences (1)–(3) above, a typical IE system would firstly detect that *Interleukin-17* and *IL-17* are phrases that describe the same gene concept and that *psoriasis* represents a disease concept. Subsequently, the system would recognise that a specific association exists between these concepts. These associations may be binary *relations* between concepts, which encode that a specific type of association exists, or they may be *events*, which encode complex *n*-ary relations between a trigger word and multiple concepts or other events. Figure 1 shows the specific characteristics of both a relation and an event using the visualisation of the brat rapid annotation tool [4]. The output of the IE system would allow the location of all sentences within a large document collection, regardless of their varied phrasing, that explicitly mention the same association, or those mentioning other related types of associations, e.g., to find different genes that have an association with psoriasis. The structured associations that are extracted may subsequently be used as input to further stages of reasoning or data mining. Many IE systems would consider that sentences (1)–(3) each conveys exactly the same information, since most such systems only take into account the key information and not the wider context. Recently, however, there has been a trend towards detecting various aspects of contextual/interpretative information (such as negation or speculation) automatically [5–8].

In this work, we focus on the automatic assignment of two interpretative *dimensions* to relations and events extracted by text mining tools. Specifically, we aim to determine whether or not each relation and event corresponds to a *Research Hypothesis*, as in sentence (1), or to *New Knowledge*, as in sentence (2). To the best of our knowledge, this work represents the first effort to apply a supervised approach to detect this type of information at such a fine-grained level.

We envisage that the recognition of these two interpretative dimensions is valuable in tasks where the discovery of emerging knowledge is important. To demonstrate the utility and portability of our method, we show that it can be used to enrich instances of both events and relations.

### Related work

The task of automatically classifying knowledge contained within scientific literature according to its intended interpretation has long been recognised as an important step towards helping researchers to make sense of the information reported, and to allow important details to be located in an efficient manner. Previous work, focussing either on general scientific text or biomedical text, has aimed to assign interpretative information to continuous textual units, varying in granularity from segments of sentences to complete paragraphs, but most frequently concerning complete sentences. Specific aspects of interpretation addressed have included negation [5], speculation [6–8], general information content/rhetorical intent, e.g., background, methods, results, insights, etc. [9–12] and the distinction between novel information and background knowledge [13, 14].

Despite the demonstrated utility of approaches such as the above, performing such classifications at the level of continuous text spans is not straightforward. For example, a single sentence or clause can introduce multiple types of information (e.g., several interactions or associations), each of which may have a different interpretation, in terms of speculation, negation, research novelty, etc. As can be seen from Fig. 1, events and relations can structure and categorise the potentially complex information that is described in a continuous text span. Following on from the successful development of IE systems that are able to extract both gene-disease relations [15–17] and biomolecular events [18, 19], there has been a growing interest in the task of assigning interpretative information to relations and events. However, given that a single sentence may contain mutiple events or relations, the challenge is to determine whether and how the interpretation of each of these structures is affected by the presence of particular words or phrases in the sentence that denote negation or speculation, etc.

IE systems are typically developed by applying supervised or semi-supervised methods to annotated corpora marked up with relations and events. There have been several efforts to manually enrich corpora with interpretative information, such that it is possible to train models to determine automatically how particular types of contxtual information in a sentence affect the interpretation of different events and relations. Most work on enriching relations and events has been focussed on one or two specific aspects of interpretation (e.g., negation [20, 21] and/or speculation [22, 23]). Subsequent work has shown that these types of information can be detected automatically [24, 25].

In contrast, work on *Meta-Knowledge (MK)* captures a wider range of contextual information, integrating and building upon various aspects of the above-mentioned schemes to create a number of separate ‘dimensions’ of information, which are aimed at capturing subtle differences in the interpretation of relations and events. Domain-specific versions of the MK scheme have been created to enrich complex event structures in two different domain corpora, i.e., the ACE-MK corpus [26], which enriches the general domain news-related events of the ACE2005 corpus [27], and the GENIA-MK corpus [28], which adds MK to the biomolecular interactions captured as events in the GENIA event corpus [22]. Recent work has focussed on the detection of uncertainty around events in the GENIA-MK Corpus. Uncertainty was detected using a hybrid approach of rules and machine learning. The authors were able to show that incorporating uncertainty into a pathway modelling task led to an improvement in curator performance [3].

The GENIA-MK annotation scheme defines five distinct *core* dimensions of MK for events, each of which has a number of possible values, as shown in Fig. 2:

1. *Knowledge Type*, which categorises the knowledge that the author wishes to express into one of: Observation, Investigation, Analysis, Method, Fact or Other.

**Fig. 2:**
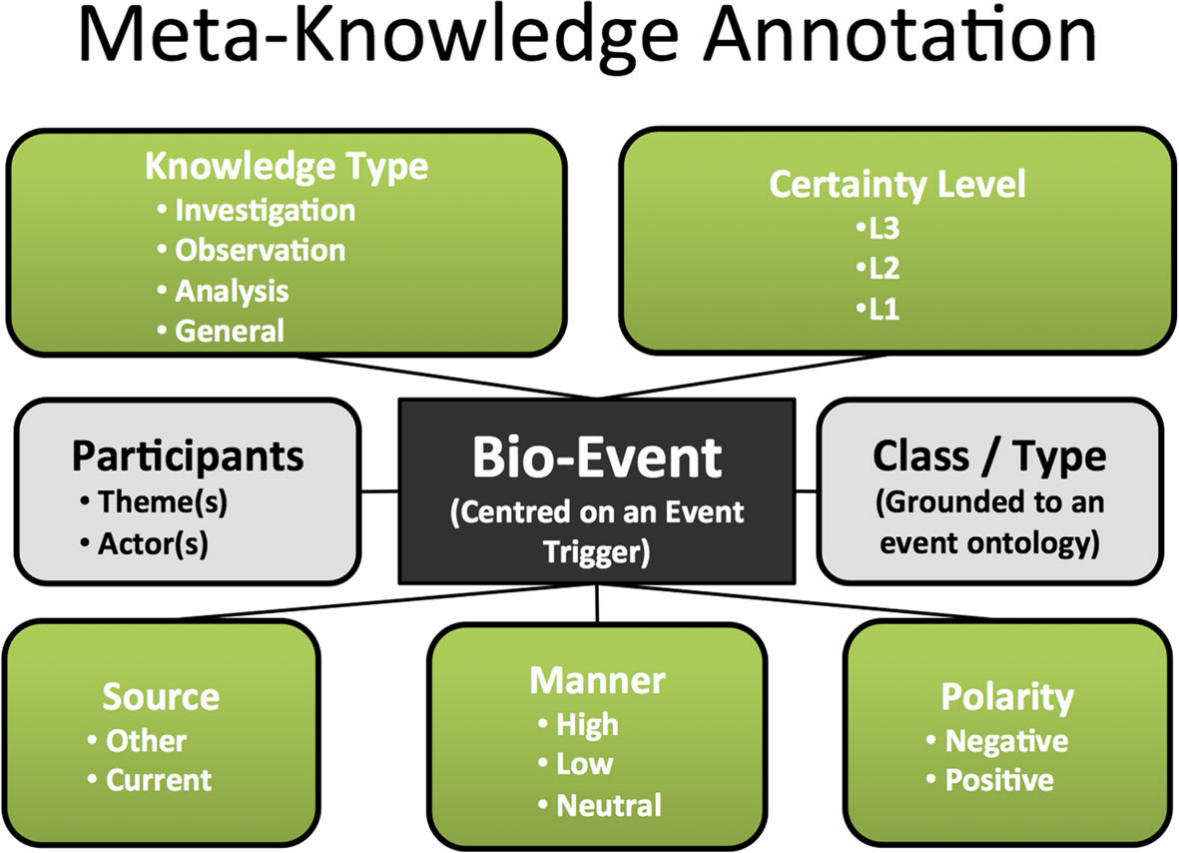
The GENIA-MK annotation scheme. There are five Meta-Knowledge dimensions introduced by Thompson et al. as well as two further hyperdimensions
2. *Knowledge Source*, which encodes whether the author presents the knowledge as part of their own work (Current), or whether it is referring to previous work (Other).
3. *Polarity*, which is set to Positive if the event took place, and to Negative if it is negated, i.e., it did not take place.
4. *Manner*, which denotes the event’s intensity, i.e., High, Low or Neutral.
5. *Certainty Level or Uncertainty*, which indicates how certain an event is. It may be certain (L3), probable (L2) or possible (L1).

These five dimensions are considered to be independent of one another, in that the value of one dimension does not affect the value of any other dimension. There may, however, be emergent correlations between the dimensions (i.e., an event with the MK value ’Knowledge Source=Other’ is more frequently negated), which occur due to the characteristics of the events. Previous work using the GENIA-MK corpus has demonstrated the feasibility of automatically recognising one or more of the MK dimensions [29–31]. In addition to the five core dimensions, Thompson et al. [28] introduced the notion of *hyperdimensions*, (i.e., New Knowledge and Hypothesis) which represent higher level dimensions of information whose values are determined according to specific combinations of values that are assigned to different core MK dimensions. These hyperdimensions are also represented in Fig. 2. We build upon these approaches in our own work to develop novel techniques for the recognition of New Knowledge and Hypothesis, which take into account several of the core MK dimensions described above, as well as other features pertaining to the structure of the event and sentence.

## Methods

Our work took as its starting point the MK hyperdimensions defined by Thompson et al. [28], since we are also interested in idenfifying relations and events that describe hypotheses or new knowledge. However, we found a number of issues with the original work on these hyperdimensions. Firstly, Thompson et al. [28] did not provide clear definitions for of ‘Hypothesis‘ and ‘New Knowledge’. In response, we have formulated concise definitions for each of them, as shown below. Secondly, by performing an analysis of events that takes into account these definitions, we found that it was not possible to reliably and consistently identify events that describe new knowledge or hypotheses based only on the values of the core MK dimensions. As such, we decided to carry out a new annotation effort to mark up both ‘Research Hypothesis’ and ‘New Knowledge’ as independent MK dimensions (i.e., their values do not necessarily have any dependence on the values of other core MK dimensons), and to explore supervised, rather than rule-based methods, to facilitate their automated recognition.

### Annotation guidelines

The starting point for our novel annotation effort was our tightened definitions of *Research Hypothesis* and *New Knowledge*; our initial definitions were refined throughout the process of annotation. As the definitions and guidelines evolved, we asked the annotators to revisit previously annotated documents in each new round. Our final definitions are presented below:

**Research Hypothesis:** A relation or event is considered as a Research Hypothesis if it encompasses a statement of the authors’ anticipated knowledge gain. This is shown in examples (1) and (2) in Table 1.

**Table 1:** Examples of sentences containing research hypotheses and new knowledge

ID	Example	Dimension
1	We **examined** the possibility of establishing new cell lines	Research hypothesis
2	We tested the **hypothesis** that oral beclomethasone dipropionate (BDP) would control gastrointestinal graft-versus-host disease…	Research hypothesis
3	These data **demonstrate** that the unexpected expression of megakaryocytic genes is a specific property of immortalized cells…	New knowledge
4	We **show** that Oral BDP prevents relapses of gastrointestinal GVHD…	New knowledge
5	CTCF is a transcriptional repressor of the c-myc gene.	—

Key words that help us to determine whether a sentence pertains to New Knowledge or Research Hypothesis are marked in bold. Some sentences may be neither Research Hypothesis nor New Knowledge, as shown in Sentence 5

**New Knowledge:** A relation or event is considered as New Knowledge if it corresponds to a novel research outcome resulting from the work the author is describing, as per examples (3) and (4) in Table 1.

Whereas the value assigned to each of the core MK dimensions of Thompson et al. is completely independent of the values assigned to the other core dimensions, our newly introduced dimensions do not maintain this independence. Rather, Research Hypothesis and New Knowledge possess the property of mutual exclusivity, as an event or relation cannnot be simultaneously both a Research Hypothesis *and* New Knowledge. We chose to enrich two different corpora with attributes encoding Research Hypothesis and New Knowledge, i.e., a subset of the biomolecular interactions annotated as events in the GENIA-MK corpus [28], and the biomarker-relevant relations involving genes, diseases and treatments in the EU-ADR corpus [23]. Leveraging the previously-added core MK annotations in the GENIA-MK corpus, we explored how these can contribute to the accurate recognition of New Knowledge and Research Hypothesis. Specifically, we have introduced new approaches for predicting the values of the core Knowledge Type and Knowledge Source dimensions, demonstrating an improvement over the former state of the art for Knowledge Type. We subsequently use supervised methods to automatically detect New Knowledge and Research Hypothesis, incorporating the values of Knowledge Type, Knowledge Source and Uncertainty as features into the trained models.

### Corpora

The GENIA-MK corpus consists of one thousand MEDLINE abstracts on the subject of transcription factors in human blood cells, which have been annotated with a range of entities and events that provide detailed, structured information about various types of biomolecular interactions that are described in text. In the GENIA-MK corpus, values for all five core MK dimensions are already manually annotated for all of the 36,000 events. The MK annotation effort also involved the identification of ‘clue words’, i.e., words or phrases that provide evidence for the assignment of values for particular MK dimensions. For example, the word ‘suggest’ would be annotated as a clue both for Uncertainty and Knowledge Type, as it indicates that the information encoded in the event is stated based on a speculative analysis of results.

The EU-ADR corpus consists of three sets of 100 MEDLINE abstracts, each obtained using different PubMed queries aimed at retrieving abstracts that are likely to contain three specific types of relations (i.e., gene-disease, gene-drug and drug-disease), the former two of which can be important in discovering how different types of genetic information influence disease susceptibility and treatment response. The original annotation task involved identifying three types of entities, i.e., targets (proteins, genes and variants), diseases and drugs, together with relationships between these entity types, where these are present. In contrast to the richness of the event representations in the GENIA-MK corpus, each relation annotation in the EU-ADR corpus consists only of links between entities of two specific types. Relations were annotated in 159 of the 300 abstracts selected for inclusion in the corpus.

### Annotation of new knowledge and research hypothesis

As an initial step of our work, subsets of GENIA-MK and EU-ADR were manually enriched with additional annotations, which identify those events or relations corresponding to Research Hypotheses or New Knowledge. Since high quality annotations are key to ensuring that accurate supervised models can be trained, we engaged with a number of experts and carried out an exploratory annotation exercise prior to the the final annotation effort, in order to ensure the highest possible inter-annotator agreement (IAA).

Initially, we worked with two domain experts, a text mining researcher and a medical professional. They added the novel MK annotations to events that had been automatically detected in sentences from full-text papers. We found, however, that there were some issues with this annotation set-up. Firstly, we found that events denoting Research Hypotheses and New Knowledge were very sparse in full papers. Secondly, we found that isolated sentences often provided insufficient context for annotators to determine accurately whether or not the event described new knowledge or a hypothesis. Finally, we found that errors in the automatically detected events were detracting the annotators’ attention from the task at hand. Based on these findings, we decided not to pursue this apporach, and instead focussed our anotation efforts on annotating Research Hypotheses and New Knowledge in abstracts containing gold-standard, expert-annotated events and relations, whose quality had previously been verified. Since abstracts also generally contain denser and more consolidated statements of New Knowledge and Research Hypotheses than full papers [32], we also expected that this approach would produce more useful training data.

We then employed two PhD students (both working in disciplines related to biological sciences) to carry out the next round of annotation work. We held regular meetings to discuss new annotations and provided feedback as necessary. A subset of the abstracts was doubly annotated by both annotators, allowing us to evaluate the annotation quality by calculating IAA using Cohen’s Kappa [33].

Table 2, which shows IAA at three different points during the annotation process, illustrates a steady increase in IAA as time progressed and as more discussions were held, demonstrating a convergence towards a common understanding of the guidelines by the two annotators. We get a final agreement of above 0.8 on most dimensions, indicating a strong level of agreement [34]. Annotation of Research Hypothesis in the EU-ADR corpus achieved slightly lower agreement of 0.761, indicating moderate agreement between the annotators [34]. At the end of the annotation process, the annotators were asked to revisit their earlier annotations to make revisions based on their enhanced understanding of the guidelines. Remaining discrepancies were resolved by the lead author after consultation with both annotators.

**Table 2:** Inter-annotator agreement across several rounds of corpus annotation as measured by Cohen’s Kappa

		Round 1	Round 2	Round 3
Research Hypothesis	EU-ADR	0.486	0.724	0.761
	GENIA-MK	0.593	0.859	0.855
New Knowledge	EU-ADR	0.627	0.825	0.842
	GENIA-MK	0.772	0.895	0.895

We show that agreement increased throughout the annotation process as we discussed difficult cases with annotators. We undertook regular meetings with the annotators to quickly resolve any disagreements

Each annotator marked up 112 abstracts from the EU-ADR corpus (70 of which were doubly annotated), and 100 abstracts from the GENIA-MK corpus (50 of which were doubly annotated). This resulted in a total of 150 GENIA-MK abstracts and 159 EU-ADR abstracts annotated with New Knowledge and Research Hypothesis. Statistics on the final corpus are shown in Table 3.

**Table 3:** Statistics comparing our versions of the GENIA-MK and EU-ADR corpora, both annotated with new knowledge and research hypothesis labels

	GENIA-MK	EU-ADR
Base type for annotations	Events	Relations
Number of annotations	6899	622
Number of abstracts	150	159
Number of new knowledge annotations	2356 (34.2%)	406 (65.3%)
Number of research hypothesis annotations	366 (5.31%)	38 (6.11%)

The GENIA-MK corpus is much more densely annotated than the EU-ADR corpus, with over ten times more annotated events in the former than annotated relations in the latter. Research Hypotheses are particularly sparse in both corpora, constituting just over 5% of all annotated relations and events in each case. There is a disparity in the proportion of New Knowledge between the two corpora, in part because the EU-ADR corpus appeared to favour the annotation of relationships denoting New Knowledge

### Baseline method for new knowledge and research hypothesis

Thompson et al. [28] suggest a method for detecting new knowledge and hypothesis based on automatic inferences from core MK values. Their inferences state that an event will be an instance of new knowledge if the Knowledge Source dimension is equal to *‘Current’*, the Uncertainty dimension is equal to *‘L3’* (equivalent to *‘Certain’* in our work, see below) and the Knowledge Type dimension is equal to either *‘Observation’* or *‘Analysis’*. Similarly, according to their inferences, an event will be an instance of Hypothesis if the Knowledge Type dimension is equal to *‘Analysis’* and Uncertainty is equal to either *‘L2’* or *‘L1’* (which are both equivalent to *‘Uncertain’* in our work, see below).

We use these automated inferences as a baseline for our techniques. To best reflect the work of Thompson et al. [28], we use their manually annotated values of Knowledge Type, Uncertainty and Knowledge Source for the GENIA-MK corpus. This allows us to compare our own work with previous efforts, as well as providing a lower bound for the performance of a rule based system, which we contrast with our supervised learning system, as introduced in the next section.

### A supervised method for extracting new knowledge and research hypothesis

We took a supervised approach to annotating events with instances of our target dimensions of New Knowledge and Research Hypothesis. According to the previously mentioned intrinsic links to the core MK dimensions of Knowledge Source, Knowledge Type and Uncertainty, we incorporated the values of these dimensions as features that are used by our classifiers.

#### Uncertainty

For the Uncertainty dimension, we used an existing system [3]. Adopting their treatment of Uncertainty, we differ from Thompson et al. [28] as we use only have 2 levels (certain and uncertain), as opposed to their three levels (L3 = certain, L2 = probable and L1 = possible). Since our development of the original MK scheme, we have experimented and discussed different levels of granularity for this dimension with domain experts, and have concluded that the differences between the two different levels of uncertainty in our original scheme (i.e., L1 and L2) are often too subtle to be of benefit in practical scenarios. Therefore, it was decided to focus instead on the binary distinction between certainty and uncertainty.

#### Knowledge source

The Knowledge Source dimension distinguishes events that encode information originating from an author’s own work (Knowledge Source = Current), from those describing work from an alternative source (Knowledge Source = Other). Such information is relevant to the identification of New Knowledge, as a relation or event that corresponds to information reported in background literature definitely cannot be classed as New Knowledge. Attribution by citation is a well-established practice in the scientific literature. Citations can be expressed heterogeneously between documents, but are typically expressed homogeneously within a single document, or a collection of similarly-sourced documents. We used regular expressions to identify citations following the work of Miwa et al. [35], in conjunction with a set of clue expressions that aim to detect background knowledge in cases where no citation is given. These include statements such as ‘we previously showed…’ or ‘as seen in our former work’. Whereas Miwa et al. use a supervised learning method to detect Knowledge Source, we found that supervised learning approaches overfitted to the overwhelming majority class (Source =Current) in the GENIA-MK dataset. This meant that we suffered poor performance on unseen data, such as the EU-ADR corpus. To alleviate this, we simply used the regular expression feature as described above as an indicator of Knowledge Source being ‘Other’. A list of our regular expressions and clue expressions is made available as part of the Additional files.

#### Knowledge type

For Knowledge Type, we used an implementation of the random forest algorithm [36] from the WEKA library [37]. We used the standard parameters of the random forest in the WEKA implementation. We used ten-fold cross validation for all experiments, and results are reported as the macro-average across the ten folds. We treat the identification of Knowledge Type as a multi-class classification problem and we took a supervised approach to categorising relations and events in the two corpora according to the values of the Knowledge Type dimension. To facilitate this, we used the following seven types of features to generate information about each event from GENIA-MK and relation from EU-ADR:

1. Sentence features describing the sentence containing the relation or event.
2. Structural features, inspired by the structural differences of events.
3. Participant features, representing the participants in the relation or event.
4. Lexical features, capturing the presence of clue words.
5. Constituency features, corresponding to relationships between a clue and the relation or event, based on the output of a parser.
6. Dependency features, which capture relationships between a clue and the relation or event based on the dependency parse tree.
7. Parse tree features, which pertain to the structure of the dependency parse tree.

These features are further described in Table 4. To generate these features, we made use of the GENIA Tagger [38] to obtain part-of-speech (POS) tags, and the Enju parser [39] to compute syntactic parse trees.

**Table 4:** Types of features used in training the Knowledge Type classification model

Feature type	Features
Sentence	SE1: length in words; SE2: length in characters; SE3: mean number of characters per word; SE4: median number of characters per word; POS tag ratios (SE5: noun-to-verb, SE6: noun-to-adjective, SE7: noun-to-adverb, SE8: verb-to-adjective, SE9: verb-to-adverb; SE10: adjective-to-adverb)
Structural	ST1: whether any participant is an event; ST2: the sentence number containing this event; ST3: whether this event is a participant in another event; ST4: whether the event is a noun phrase; ST5: whether the event is an instance of “regulation”; ST6: total number of themes; ST7: total number of causes
Participant	PA1: POS tag of the first participant; PA2: POS tag of the first cause; PA3: whether any theme is an event; PA4: whether any cause is an event; PA5: POS tag of the word in a governing dependency over the theme; PA6: POS tag of the word in a governing dependency over the cause
Lexical	L1: distance between nearest clue and event trigger; L2: whether sentence contains at least one clue; L-N which clues (in a precompiled list) are matched within the sentence; features of matched clue (L3: surface form, L4: POS tag, L5: position relative to trigger, L6: whether in auxiliary form); L7: whether trigger contains a cue; features of nearest clue (L8: tense, L9: aspect, L10: voice); L11-L15: whether clue usually occurs in the context of each Knowledge Type; L16: number of matched clues;
Constituency	Relationships between clue and event trigger (C1: s-commands, C2: vp-commands, C3: np-commands); relationships between clue and any event participant (C4: s-commands, C5: vp-commands, C6: np-commands); C7: whether scope of any clue is within the same scope as the trigger
Dependency	Direct dependencies (D1: between clue and trigger, D2: between clue and any event participant); one-hop dependencies (D3: between clue and trigger, D4: between clue and any event participant); two-hop dependencies (D5: between clue and trigger, D6: between clue and any event participant)
Parse Tree	Distances: PT1: between theme and furthest leaf node; PT2: between cause and furthest leaf node; PT3: between theme and root node; PT4: between cause and root node

A detailed explanation of each feature with examples is given in the Additional files

#### Research hypotheses and new knowledge

We followed a similar approach to predicting Research Hypothesis and New Knowledge values to that described above for the recognition of Knowledge Type. We used the same features and also a random forest classifier. We incorporated additional features encoding the Knowledge Source, Knowledge Type and Uncertainty of each relation and event.

Clue lists, developed by the authors, were used for the detection of Knowledge Type, Knowledge Source and Uncertainty. For the detection of New Knowledge and Hypothesis, a combination of clues for Knowledge Type, Knowledge Source and Uncertainty was used. The exact clue lists are available in the Additional files.

## Results

In this section, we present our experiments to detect the core Knowledge Type dimension, in which we determine the most appropriate feature subset to use, and also compare our approach to previous work. We then extend this approach to recognise New Knowledge and Research Hypothesis, and to evaluate our results in terms of *precision*^1^, *recall*, 2 and *F1-score*. 3

Our experiments to predict the correct values for the Knowledge Type dimension were carried out only using the events in the GENIA-MK corpus, given that Knowledge Type is only annotated in this corpus and not in EU-ADR. We performed an analysis of each feature subset to assess its impact on classifier performance, as shown in Table 5. It was established that removing each of the participant, dependency and parse tree features individually leads to a small increase in F1-score. However, in subsequent experiments, we found that removing all three features does not lead to an additional increase in performance. We therefore used all feature subsets except for the participant features in subsequent experiments, as this gave us the best overall score. By observing the isolated performance of each feature subset, we also determined that the lexical and structural features are both significant individual contributors to the final classification score. In Table 6, we compare the performance of our classifier in predicting each Knowledge Type value with the results obtained by the state-of-the-art method developed by Miwa et al. [31]. The results reveal that our approach achieves an increase in F1-score over Miwa et al. [31] by a minimum of 0.063 for the Other value, and a maximum of 0.113 for Method. We also see corresponding performance boosts in terms of precision and recall. Although we observe a small drop in recall for Fact and Method, this is offset by an increase in precision of 0.210 and 0.299, respectively.

**Table 5:** Effects of each feature subset on the final classification performance for Knowledge Type

Feature Subset	Only This Feature			All Except This Feature		
	P	R	F1	P	R	F1
Constituency	—	—	—	0.815	0.727	0.763
Dependency	—	—	—	0.823	0.728	0.765
Parse Tree	0.428	0.281	0.340	0.823	0.730	**0.776**
Participant	0.383	0.252	0.243	**0.831**	**0.740**	**0.776**
Sentence	0.474	0.442	0.453	0.785	0.705	0.738
Lexical	**0.592**	0.449	0.478	0.794	0.722	0.754
Structural	0.558	**0.495**	**0.517**	0.791	0.665	0.709
All	0.823	0.725	0.764	0.823	0.725	0.764

Results are only shown in cases where it was possible to produce a reliable model. The final row denotes the performance of the classifier when using all feature subsets

Values in bold represent the best performing feature subset for each column

**Table 6:** A comparison of the Knowledge Type results produced by our classifier against the results of the most directly comparable work

Knowledge Type	RF — our features			Miwa et al. 2012 [31] (SVM)			SVM — our features		
	P	R	F1	P	R	F1	P	R	F1
Observation	0.781	0.853	0.815	0.721	0.723	0.722	0.658	0.744	0.698
Fact	0.847	0.648	0.734	0.637	0.680	0.658	0.506	0.310	0.384
Other	0.788	0.810	0.799	0.770	0.706	0.736	0.727	0.671	0.698
Method	0.832	0.535	0.651	0.534	0.543	0.538	0.641	0.455	0.532
Investigation	0.884	0.763	0.819	0.691	0.755	0.722	0.724	0.714	0.718
Analysis	0.852	0.826	0.838	0.704	0.784	0.742	0.718	0.793	0.754

To enable a more direct comparison, we have also provided our results when using a SVM (the classifier used by Miwa et al.) with our features

To further investigate our improvement over Miwa et al., we swapped our classifier for an SVM, but used all the same features. The results of this are shown in Table 6. This experiment allowed us to compare the performance of our features with the same classification algorithm (SVM), as used by Miwa et al. We note that using the SVM with our features leads to a similar, but slightly worse performance in terms of F1 score than Miwa et al. on all categories except for Analysis. However we do note an increase in Precision for certain categories (Method, Investigation, Analysis) and Recall for others (Observation, Analysis). As our features are tuned for performance with a Random Forest, this experiment demonstrates that different types of classifiers may require different feature sets to achieve optimal performance.

To further understand the impact of our feature categories, we analysed the correlation of each feature with each Knowledge Type value. This allowed us to determine the most informative features for each Knowlegde Type value, as displayed in Table 7. In addition to this, we calculated the average rank of each feature across all Knowledge Type values. This measure shows us the most globally useful features. The top features according to average rank are displayed in Table 8.

**Table 7:** The top-10 most informative features for each Knowledge Type value

#	Observation		Fact		Other		Method		Investigation		Analysis	
1	C7	0.313	ST3	0.173	ST3	0.487	ST5	0.203	L-47	0.308	C5	0.364
2	L5	0.263	ST2	0.154	ST1	0.330	ST1	0.135	L-46	0.292	L11	0.343
3	L11	0.255	ST5	0.110	ST5	0.216	L-48	0.100	ST4	0.227	C4	0.301
4	C2	0.252	C7	0.097	L11	0.131	ST3	0.075	L13	0.221	ST3	0.283
5	C5	0.218	L2	0.088	C7	0.127	C7	0.063	ST2	0.209	C2	0.258
6	L16	0.211	L5	0.076	L5	0.119	L14	0.060	ST3	0.202	C7	0.257
7	C1	0.207	L11	0.068	D1	0.108	L9	0.056	SE5	0.195	D1	0.234
8	L2	0.196	SE10	0.064	SE3	0.096	C5	0.051	D1	0.151	ST1	0.227
9	C2	0.178	L-35	0.063	L-28	0.090	SE1	0.046	D2	0.144	C1	0.203
10	L15	0.173	C1	0.061	L2	0.087	C4	0.045	L11	0.141	L5	0.197

These were calculated using Pearson’s correlation between each class label and each feature. The feature labels are expanded in Table 4, above

**Table 8:** The 10 top ranked features, averaged across all classes for Knowledge Type

#	Feature	Average Rank
1	C7	5.50
2	L11	6.17
3	ST3	8.33
4	L5	9.17
5	ST1	11.33
6	C4	12.50
7	D1	14.17
8	ST5	14.67
9	C5	15.33
10	L-5	18.50

This shows which features are globally informative. The feature labels are expanded in Table 4, above

For the identification of New Knowledge and Research Hypothesis, we firstly performed 10-fold cross validation on each corpus (GENIA-MK and EU-ADR) and for each dimension of interest, yielding the results in Table 9. In our presentation of results, we term the negative class for New Knowledge as “Other Knowledge”, as it covers a number of categories that we wish to exclude (e.g., background knowledge, irrelevant knowledge, supporting knowledge, etc.). We were able to classify Knowledge Type for relations in the EU-ADR corpus by setting the event and participant features to sensible static values — e.g., the number of participants in a relation is always 2.

**Table 9:** Results of 10-fold cross validation on both datasets for Research Hypothesis and New Knowledge

			P	R	F1
GENIA-MK	Majority Baseline	New Knowledge	0.000	0.000	0.000
		Other knowledge	0.659	1.000	0.794
		Average	0.329	0.500	0.397
		Hypothetical	0.000	0.000	0.000
		Non-Hypothetical	0.947	1.000	0.973
		Average	0.473	0.500	0.486
	Rule-based Baseline	New Knowledge	0.580	0.767	0.660
		Other knowledge	0.855	0.712	0.777
		Average	0.717	0.739	0.719
		Hypothetical	0.054	0.077	0.063
		Non-Hypothetical	0.947	0.924	0.936
		Average	0.500	0.500	0.499
	Random Forest	New Knowledge	0.863	0.920	0.891
		Other knowledge	0.823	0.719	0.767
		Average	0.843	0.819	0.829
		Hypothetical	0.928	0.762	0.836
		Non-Hypothetical	0.987	0.997	0.992
		Average	0.958	0.880	0.914
EU-ADR	Majority Baseline	New Knowledge	0.644	1.000	0.784
		Other knowledge	0.000	0.000	0.000
		Average	0.322	0.5	0.392
		Hypothetical	0.000	0.000	0.000
		Non-Hypothetical	0.939	1.000	0.968
		Average	0.469	0.500	0.484
	Random Forest	New Knowledge	0.853	0.921	0.884
		Other knowledge	0.831	0.692	0.748
		Average	0.842	0.807	0.816
		Hypothetical	1.00	0.533	0.668
		Non-Hypothetical	0.970	1.00	0.9848
		Average	0.985	0.767	0.827

We report precision (P), recall (R) and F1-score. In each major row below, the first two sub-rows represent the macro average of 10-fold cross validation on each class. The third sub-row represents the average of the two classes above it. We have included a majority class baseline below for comparison. This was calculated by assigning every event to the majority class and then calculating the results of precision, recall and F1 score. The majority class is the negative class for both New Knowledge and Hypothesis in the GENIA-MK corpus. In the EU-ADR corpus, the majority class is the positive class for New Knowledge and the negative class for Hypothesis. In addition, we include results for the rule-based baseline from Thompson et al. [28], as described previously

## Discussion

In Table 5, we observed the effects of each feature subset on the overall classification score for Knowledge Type. We found that the structural, lexical and sentence features had particularly strong contributions. The structural features encoded information about the structure of the event and were particularly useful for identifying events that participate in other events. The lexical features depended on the identification of clue words that appeared in the context of relations and events, which provided important evidence to determine the most appropriate MK values to assign. However, the usefulness of this feature is directly tied to the comprehensiveness of the list of clues associated with each MK value.

In addition to the feature analysis in Table 5, we also provided additional analysis of each specific feature in Tables 7 and 8. In line with the results from Table 5, these tables demonstrate that the structural features were particularly informative for most classes, as well as the lexical, dependency and constituency features. It is interesting to note from Table 7 that no individual feature is particularly strongly correlated with each class label. This supports our ensemble approach and indicates that multiple feature sources are needed to attain a high classification accuracy. In addition, we can see that the correlations drop fairly quickly for all classes - indicating that not all features are used for every class. Finally, we can see that different features occur in each column (with some repetition), indicating that certain features were more useful for specific classes.

For the classification of New Knowledge and Hypothesis, we incorporated features denoting the existing meta-knowledge values of the event for Knowledge Source, Knowledge Type and Uncertainty. Knowledge Source indicates whether an event is current to the research in question, or whether it describes background work. This may be especially helpful for the detection of new knowledge, since it is clear that any background work cannot be classified as new knowledge. Knowledge Type classifies events as falling into one of six categories, i.e., Fact, Method, Analysis, Investigation, Observation or Other. The Investigation category may have contributed to the classification of Hypothetical events, whereas Observation and Analysis may have helped to contribute to the detection of New Knowledge events. The Fact, Method and Other categories could have helped the system to determine that events did not convey either hyperdimension. Finally, Uncertainty describes whether an author presented their results with confidence in their accuracy, or with some hedging (e.g., use of the words *may, possibly, perhaps*, etc.). This dimension could have helped to contribute to the classification of hypotheses (where an author states that an event may occur) and new knowledge, where we expect an author to be certain about their results.

We compared our results to those of Miwa et al. (2012) in Table 6, where we showed a consistent improvement of precision, recall and F1-score across all categories. Their system used support vector machines (SVMs) for classification, with a set of features similar to our lexical and structural features. However, our work used an enhanced set of features as well as a random forest classifier, which is typically robust in high dimensional classification problems [36]. These two factors contributed to our system’s improved performance. Our system yielded an average increase in precision of 0.156, but only yielded an average increase in recall of 0.04. This implies that the use of a random forest and additional features mainly helped to ensure that the system returned results which are consistently correct. For both the ‘Fact’ and ‘Method’ Knowledge Type values, our system yielded a slight dip in recall compared to previous work. However, this was coupled with an increase in precision of 0.210 and 0.298, respectively.

To understand the relative contributions made by our switches in both feature set and type of classifier, compared to previous work, we analysed the performance of our system when using an SVM with our features instead of a Random Forest. We attained a similar performance to Miwa et al. using our feature set and SVM, although some values were lower than those reported by Miwa et al. This implies that our decision to use a different type of classifier to Miwa et al. (i.e., Random Forest instead of SVM) was the main reason behind our improved performance. Different feature sets are better suited to different types of classifiers, and our feature set was carefully selected (as documented in Table 5) to be performant with a Random Forest. Miwa et al.’s features were equally selected to perform well with an SVM. We have shown similar results in prior work for a task on detecting metaknowledge for negated bio-events [29], where we showed that tree-based methods, including the Random Forest, outperformed other techniques such as the SVM for detecting the negation dimension of metaknowledge.

We illustrated our results for the identification of the novel dimensions New Knowledge and Research Hypothesis in Table 9. These showed strong performance across both corpora and association types (events and relations). The results for the GENIA-MK corpus (events) outperformed those for the EU-ADR corpus (relations). This was most likely due to the difference in size between the corpora. There are over ten times more annotated events in the subset of GENIA-MK that we annotated than relations in the subset of EU-ADR (6899 events vs. 622 relations). The fact that we annotated all of the 159 abstracts available in the EU-ADR corpus and only 150 abstracts from GENIA-MK indicates that event structures are more densely packed in GENIA-MK than relations in EU-ADR.

In particular, the EU-ADR corpus yielded a poor recall value for Research Hypotheses. There were only 38 examples of relations annotated as Research Hypothesis in the EU-ADR corpus. Our annotators reported that several relations occuring in hypothetical contexts appeared to have been missed by the original annotators of the EU-ADR corpus, which may be the cause of this sparsity. However, adding additional relations to the corpus was beyond the scope of the current work. The precision for the prediction of Research Hypothesis in the EU-ADR corpus was 1.00, indicating that of those relations automatically classified as Research Hypothesis, all were indeed Research Hypotheses (i.e., there were no false positives). It is usually the case in minority class situations that a classifier will tend towards classifying instances as the majority class (i.e., favouring false negatives over false positives), so this result is expected. We chose not to perform subsampling of the majority class, as the density of Research Hypotheses or New Knowledge in our training data is reflective of the density we would expect in other biomedical abstracts.

Our corpus has focussed on identifying Research Hypotheses and New Knowledge in biomedical abstracts. However, it has been shown elsewhere that full texts contain more information than abstracts alone [40]. Whilst our future goal is to additionally facilitate the recognition of New Knowledge and Research Hypothesis in full papers, our decision to focus initially on abstracts was motivated by the findings of our earlier rounds of annotation. These initial annotation efforts revealed that the density of the types of MK that form the focus of the current paper are very low in full papers and are consequently difficult for annotators to reliably identify. Therefore we chose to use abstracts, where the density was higher, since the availability of as many examples as possible of relevant MK was important for the development of our methods. We noted that abstracts fairly consistently mention the main Research Hypotheses and New Knowledge outcomes from a paper. However, further information may be available in the full paper that has not been mentioned in the abstract. To access this information we will need to further adapt our techniques and develop annotated corpora of full papers — this is left for future work.

### Error analysis

Finally, we present an analysis of some common errors that our system makes and strategies for overcoming these in future work. In the following sentence, the event centred on “regulation” was marked as Non-Hypothetical by the annotators, but our system recognised it as a Hypothetical event.

*To continue our investigation of the cellular events that occur following human CMV (HCMV) infection, we* **focused** *on the* **regulation** *of cellular activation following viral binding to human monocytes.*

**Table:** 

**Event ID:**	E1
**Trigger:**	regulation
**Theme:**	activation following viral binding
**Cause:**	N/A
**Clue:**	focused

It is likely that this event was marked as a hypothesis by the system because of the words ‘investigation’ and ‘focused’ that occur before it. However in this case, the main hypothesis that the annotators have marked is on the event centred on ‘occur’ preceding the event centred around ‘focused’. To overcome this in future work, we could implement a classification strategy that takes into account MK information that has already been assigned to other events that occur in the context of the focussed event. A conditional random field or deep learning model could be used for sequence labelling to accomplish this.

The second error, which concerns the event centred on “effects” in the following sentence, was marked as Hypothetical by our annotators, but was classified as Non-Hypothetical by our system.

*MATERIAL AND METHODS: In the present study, we analyzed the* **effects** *of CyA, aspirin, and indomethacin* $\dots $

**Table:** 

**Event ID:**	E2
**Trigger:**	effects
**Theme:**	Cya, aspirin, and indomethacin
**Cause:**	N/A
**Clue:**	present study

This event is clearly stating the subject of the authors’ investigation, and so should be marked as hypothesis. It is likely that our system was confused by the preceding section heading, which led it to believe that this was part of the background or methods, and not a statement of the authors’ intended research goals. To overcome this, we could identify these section headings automatically and either exclude them from the text to be analysed, or use them as extra features in our classification scheme.

In our third example error, the event in the sentence below is centred on the phrase “result in decreased”. The event was marked as new knowledge by the annotators, but the system was not able to recognise it as such.

*Down-regulation of MCP-1 expression by aspirin may* **result in decreased** *recruitment of monocytes into the arterial intima beneath stressed EC.*

**Table:** 

**Event ID:**	E3
**Trigger:**	result in decreased
**Theme:**	recruitment of monocytes
**Cause:**	Down-regulation of MCP-1 expression
	by aspirin
**Clue:**	N/A

We believe that the cause of this classification errors is the unusual event trigger - the majority of events only have a single verb as their trigger. To help the system to better determine cases in which such events denote new knowledge, it would be necessary to further increase our corpus size, such that the training set includes a wider variety of trigger types. A further factor affecting the inability of the system to determine the new knowledge classification may have been be the lack of an appropriate new knowledge clue. In this case, the annotators most likely determined this as an example of new knowledge due to information from the wider context of the discourse. We could improve our classifier by looking for clues in a wider window, or by looking for discourse clues that might indicate that the author is drawing their conclusions.

The final example below concerns an event (centred on the verb “enhanced”), which was marked as ‘other knowledge’ by the annotators, but which the system determined to be an example of new knowledge.

*Taken together, these data indicate that the unexpected* **expression** *of megakaryocytic genes is a specific property of immortalized cells that cannot be explained only by* **enhanced** *expression of Spi-1 and/or Fli-1 genes*

**Table:** 

**Event ID:**	E4
**Trigger:**	expression
**Theme:**	megakaryotic genes
**Cause:**	N/A
**Clue:**	indicate
**Event ID:**	E5
**Trigger:**	enhanced
**Theme:**	expression of Spi-1 and…
**Cause:**	E4
**Clue:**	N/A

In this example, the event is somewhat problematic as regards the assignment of MK. Although it is clear both that the sentence is a concluding statement, and that there is some new knowledge contained within it, the annotators chose not to mark the event with the trigger “enhanced” as new knowledge, indicating that they did not consider it to convey the main aspect of new knowledge in this sentence. Interestingly, however, both annotators agreed with the system that the event centred on the first instance of “expression” should be marked as an instance of new knowledge. The presence of the clue ‘indicate’ may be affecting the system’s classification decision in both cases. A human annotator can distinguish that indicate is most relevant to ‘expression’, rather than ‘enhanced’, whereas our system was unable to make this distinction.

## Conclusions

We have presented a novel application of text mining techniques for the discovery of Research Hypotheses and New Knowledge at the level of events and relations. This constitutes the first study into the application of supervised methods to assign these interpretative aspects at such a fine-grained level. We firstly showed that by applying a Random Forest classifier using a new feature set, we were able to achieve a better performance than previous efforts in detecting Knowledge Type. We subsequently showed that the core MK dimensions of Knowledge Type, Knowledge Source and Uncertainty could feed into the training of classifiers that can predict whether events and relations represent Research Hypotheses and New Knowledge, with a high degree of accuracy. Our techniques can be incorporated into a system that allows researchers to quickly filter information contained within the abstracts of research articles, as shown in previous literature [3]. Our methods generally favour precision on the positive class (i.e., Research Hypothesis or New Knowledge). Specifically, we attain a precision of between 0.863 and 1.00 on all of the corpus experiments. This demonstrates that our approach is successful in avoiding the identification of false positives, thus allowing researchers to be confident that instances of Research Hypothesis or New Knowledge identified by our method will usually be correct.

## Additional files

Additional file 1 The annotation guidelines that were given to annotators for reference. (PDF 830 kb)

Additional file 2 A table providing an in depth description of each feature. (PDF 32 kb)

Additional file 3 Read me documentation explaining the structure of the clue files. (TXT 4 kb)

Additional file 4 The clues used to detect the Analysis component of the Knowledge Type meta-knowledge dimension. (FILE 3 kb)

Additional file 5 The clues used to detect the Fact component of the Knowledge Type meta-knowledge dimension. (FILE 4 kb)

Additional file 6 The clues used to detect the Investigation component of the Knowledge Type meta-knowledge dimension. (FILE 2 kb)

Additional file 7 The clues used to detect the Method component of the Knowledge Type meta-knowledge dimension. (FILE 4 kb)

Additional file 8 The clues used to detect the Observation component of the Knowledge Type meta-knowledge dimension. (FILE 4 kb)

Additional file 9 The clues used to detect the Other component of the Knowledge Source meta-knowledge dimension. (FILE 1 kb)

Additional file 10 The clues used to detect the Uncertain component of the Certainty Level meta-knowledge dimension. (FILE 4 kb)

## Abbreviations

ADR: Adverse Drug Reaction
F1: F1 Score (The harmonic mean between Precision and Recall)
IE: Information Extraction
IAA: Inter-Annotator Agreement
MK: Meta-Knowledge
P: Precision
R: Recall
SVM: Support Vector Machine
TM: Text Mining

## Footnotes

1 the proportion of results returned by the system which are correct.

2 the proportion of correct results returned by the system as a fraction of all the correct results that should have been found.

3 the balanced harmonic mean between precision and recall, providing a single overall measure of performance.
