# Supplementary material for: Identification of research hypotheses and new knowledge from scientific literature
Source: BMC Med Inform Decis Mak. 2018 Jun 25;18:46. doi: 10.1186/s12911-018-0639-1 (PMC6019216; doi:10.1186/s12911-018-0639-1)
Supplement: Supplementary file 1 — The annotation guidelines that were given to annotators for reference. (PDF 830 kb) [file 12911_2018_639_MOESM1_ESM.pdf]

Meta-Knowledge Annotation of Bio-Events

Annotation Guidelines

Table of Contents

TABLE OF CONTENTS 2

1 INTRODUCTION AND BACKGROUND 3

1.1 BACKGROUND TO THE TASK –SEARCHING FOR RELEVANT INFORMATION 3
1.1.1 KEYWORD SEARCHING AND ITS PROBLEMS 4
1.1.2 EVENTS AND EVENT-BASED SEARCHING 4
1.2 NEED FOR META-KNOWLEDGE ANNOTATION 6
1.2.1 META-KNOWLEDGE EXAMPLES 7

2 THE ANNOTATION SCHEME 9

2.1 NEW KNOWLEDGE 9
2.1.1 NEW KNOWLEDGE 9
2.1.2 IRRELEVANT KNOWLEDGE 10
2.2 HYPOTHESIS 10
2.2.1 HYPOTHETICAL 10
2.2.2 NON-HYPOTHETICAL 11

3 EXAMPLES 12

4 ANNOTATION TASK 14

4.1 WHAT ANNOTATION IS ALREADY THERE? 14
4.2 WHAT DOES THE ANNOTATION TASK INVOLVE? 14

5 ANNOTATION ENVIRONMENT 15

5.1.1 USER LOGIN 15
5.1.2 DOCUMENT NAVIGATION 15
5.1.3 META-KNOWLEDGE ANNOTATION 16
5.1.4 PROVIDING POINTS FOR DISCUSSION 16

1 Introduction and Background

If a user wishes to search for relevant information located within biomedical documents, the usual method is to enter keywords into a search engine. However, such searches normally return a large number of documents, many of which are likely to be irrelevant.

Assume that the user wishes to find instances of positive regulations involving the protein *narL gene product.* He may enter the search terms "*narL gene product"* and *activate*, since instances of positive regulations are often described using the verb *activate.* Although his goal is to find documents where these search terms are related to each other in a specific way, the problem is that normal search engines do not take account of relationships between search terms, and may even return documents where the 2 search terms are each located in a separate sentence.

Text mining systems help to cut down on the amount of time that users have to spend sifting through irrelevant documents. This is facilitated by providing the user with the means to formulate more structured queries, which ensure that only those documents containing the required type of knowledge are returned by the search. Using a text mining system, the user can specify that he wishes to find all instances of positive regulations, where *the narL gene product* is the instigator of the regulation. It is not necessary to worry about exactly how the regulation is expressed in the text, e.g., which verb is used.

Although text mining systems providing functionality such as the above have already been developed, what they often lack is a means to distinguish between definite facts and other types of interpretations. For example, a text mining system may retrieve the following fact in response to the query above:

(S1) The narL gene product activates the nitrate reductase operon

Sentence (S1) can fairly certainly be interpreted as describing a definite fact. However, compare this to sentence (S2):

(S2) Our results suggest that the narL gene product activates the nitrate reductase operon

In (S2), the first part of the sentence projects a rather different interpretation to the information described by the verb *activates*, i.e., it is a somewhat tentative interpretation/analysis of results, which should certainly not be interpreted as a definite fact.

The ability to distinguish between different interpretations of information can be important, e.g., a biologist may want to search a collection of documents to isolate descriptions of *new knowledge* (e.g., experimental observations and confident analyses of results) from other types of knowledge (e.g., descriptions of well-established knowledge, hypotheses, etc.). This could be useful, for example, in maintaining an up-to-date database of biological interactions. If the isolation of new knowledge from other types of knowledge can be carried out automatically, this can potentially save the user a large amount of time.

In order to produce systems that can distinguish different interpretations of information, we need to undertake a task called *annotation.* This involves reading texts and identifying and marking (annotating) the different ways in which information relating to the interpretation of knowledge (which we term *meta-knowledge*) can be expressed in texts. The text mining system can then learn to generalize from the annotated examples (using a computer algorithm), in order to be able to assign interpretation information to previously unseen examples. This annotation process is the subject of this document.

1.1 Background to the Task –Searching for Relevant Information

Complex, structured queries such as those introduced above must be matched against structured representations of the biological knowledge that occurs in documents. Text mining systems need to be able to analyse texts in order to locate this biological knowledge and produce structured representations from the unstructured text. These structured representations of knowledge are called *events.* A number of collections of documents (called corpora) contain event annotations. These have been produced by domain experts, in order to allow text mining systems to learn how to recognise relevant events within texts. The meta-knowledge annotation introduced above will be carried out for individual events within these event-annotated corpora. This will provide the necessary information to train systems which not only recognise events, but can also determine automatically how those events should be interpreted.

In this section, we firstly look more closely at why events and event-based searching are needed, by examining the more usual keyword searches, and highlighting their pitfalls. We then move on to look at an example of an event, and how searching using events can be more powerful and can retrieve more focussed results than are possible using keyword searches

1.1.1 Keyword Searching and its Problems

It is often necessary for biologists to search the literature for relevant information. For example, a particular user may be interested in discovering the types of things that are positively regulated by a particular protein, e.g. *the narL gene product.* A sentence such as (S1) would provide the type of information that is sought:

(S1) The narL gene product activates the nitrate reductase operon

In other words, one type of sentence that would help the user to locate the information they require would be one in which *The narL gene product* is the grammatical subject of a verb which describes a positive regulation (such as *activate*). In such a sentence, the grammatical object of the verb (i.e., *the nitrate reductase operon* in the above example) will provide the information that is sought.

As mentioned above, using a search engine such as *Google* or *PubMed* would involve entering keywords and phrases such as *"narL gene product"* and *"activate"*. Although a search carried out using these terms is highly likely to retrieve relevant documents, it is just as likely to retrieve a large number of documents that are not relevant.

Keyword searches such as the above can be problematic for a number of reasons, and can retrieve many irrelevant documents as well as relevant ones. For example:

- Searching for *The narL gene product* and *activate* as separate search terms does not guarantee that they will be grammatically related to each other in the text in the way specified above. The search terms may not even occur within the same sentence.
- Searching using a single quoted search term, e.g., "*The narL gene product activates"*, to ensure that the verb occurs next to the protein in the text, is also not sufficient. The set of documents returned by such a query is likely to be smaller and more relevant than if using separate search terms. However, many relevant documents could also be missed, due to the large number of potential variations in the way that the positive regulation can be expressed in text. Some similar phrasings of the sentence (1) would include "*The narL gene product* ***is known to*** *activate the nitrate reductase operon.",* "*The narL gene product* ***rapidly*** *activates the nitrate reductase operon",* "*The nitrate reductase operon* ***is activated by*** *the narL gene product".*
- Positive regulation events may be described by a number of different verbs and nouns other than activate e.g. *increase, affect, effect*

In short, retrieving all relevant documents using simple keyword searches can be rather time consuming, and will often require a number of separate searches to be carried out, and much sifting of the documents returned in order to distinguish those documents that are relevant to the query.

1.1.2 Events and Event-Based Searching

Text mining technology can help greatly in searching for information, both to giving extra power to the searching mechanism, thus reducing the number of separate searches that have to be carried out, as well as increasing the relevance of the results that are returned by the search.

Unlike traditional search engines, text mining systems do not simply view documents as sequences of words, but rather they try to *structure* this information automatically, and try to find relationships between words and phrases within sentences. These structures are called *events* and the automatic process is called *event extraction.*
A possible structured representation of the event described in sentence (S1) would be the following:

EVENT_TYPE: *Positive_Regulation*
EVENT_TRIGGER: *activates*
CAUSE: *The narL gene product* (PROTEIN)
THEME: *the nitrate reductase operon* (OPERON)

The main features of this representation are as follows:

- EVENT_TRIGGER – a word or phrase around which the event is "organized" in the text. This is often a verb (in this case *activates*) or nominalized verb (a noun with a verb-like meaning, such as *transcription* or *activation)*
- EVENT_TYPE - The event is assigned a type from a fixed set of possible values that characterise different types of events in biomedical texts. The event type abstracts away from the actual verb used to describe the event in the text.
- Event participants – Each event has one or more participants. These are generally entities (e.g. genes, proteins, organisms, etc.) that play a part in description of the event. Each participant is separately identified and assigned the following information:
  - Semantic role – a label that characterizes the contribution of the participant towards the description of the event. The labels used are rather general, as they are intended to be applicable to all events in biomedical texts. The following roles are used in the description above.
    - CAUSE – participant responsible for the event occurring
    - THEME – participant affected by or during the event
  - Named Entity (NE) type – a label that characterizes the type of biological entity that the event participant represents (e.g. PROTEIN). Again, these types are chosen from a fixed set of values.

The automatic extraction of such events from texts allows searches to be carried out on these structures themselves, rather than using keyword searches on the unstructured text. The event structure abstracts from the exact wording in the text, meaning that searches over events can specify the following:

- Event types (e.g. *Negative_regulation, Binding*) instead of precise verbs or nominalised verbs used to describe the event
- Restrictions on the event participants in terms of:
  - Semantic roles specified by the event (e.g., CAUSE, THEME)
  - Values of particular roles, which could be specified as either:
    - Keywords when searching for specific values (e.g., *narL gene product*)
    - NE types for a more general search (e.g. events where the CAUSE is any entity of type PROTEIN)

Thus, the user has a choice about how general or specific to make their query. NE and event types are often arranged into a hierarchy, giving the use even more control over how general or specific their search will be.

As event-based searching allows users to be more precise about the type of information they are looking for, the set of results is better aligned with the users requirements, i.e., the results are more focussed, and contain fewer irrelevant documents than simple keyword searches. The results are also more concise than those returned by a traditional search engine, showing only the relevant events, or the sentences from the documents in which the relevant events are contained, rather than complete documents.
In more complex sentences, it is possible for multiple events to be present, and it is also possible for the participant of a particular event to be another event. Consider example (S3).

(S3) We found that Y activates the expression of X

Here, the "main" event in the sentence, i.e., the one which is triggered by the verb *activates*, has a similar structure to the event in sentence (S1), except that the *THEME* of the event (i.e. *the expression of X*) is not a simple entity, so how do we deal with it?

EVENT_TYPE: *Positive_Regulation*
EVENT_TRIGGER: *activates*
CAUSE: *Y*
THEME: *?*

We actually treat this THEME as being a separate event, as it can be seen as having its own structure, with the type *GENE_EXPRESSION* and the *THEME* of X. Note that is not necessary for both CAUSE and THEME to be specified for all events. To deal with the fact that this second event is a participant of the first, we assign the unique identifiers *E1* and *E2* to the events. Figure 1 shows the full representation of these 2 events.

Using this notation, the biological knowledge contained in a document can be represented a set of events, some of which will be "nested" within each other.

We refer to E2 as a primary event, and E1 as a secondary event. E2 conveys the main information, whilst E1 can be seen as providing supporting information – it is not a complete or "interesting" piece of knowledge in itself. It is often (but not exclusively) the case that primary events have event triggers that are verbs, whilst secondary events have triggers that are a special type of noun with a verb-like meaning called *nominalised verbs.* The noun *expression* is an example of one of these, with a meaning similar to the verb *express.* Other examples would include *transcription* (from the verb *transcribe*) and *regulation* (from the verb *regulate*).

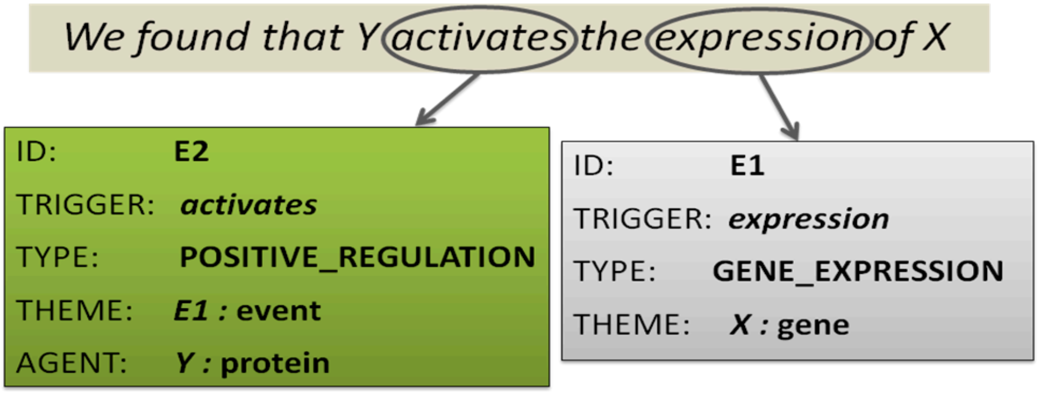

Figure 1 – Event Representation Example

1.2 Need for Meta-Knowledge Annotation

Text mining systems are normally trained to recognise events by learning from annotated examples. That is to say, a collection of documents (called a *corpus*, plural *corpora*) are annotated with events by human domain experts. The event annotation process often involves:

- Locating the event trigger
- Assigning a type to the event
- Identifying the participants of the event
- Assigning roles and NE types to these participants

In the biomedical field, a number of such annotated corpora already exist, making it possible to train systems to recognize events and their participants. However, information about the *interpretation* of the events (i.e., meta-knowledge) is often missing from the annotation, or it is not dealt with in a satisfactory way.

Some examples of meta-knowledge that we consider to be important include the following:

- Is the event stated as a hypothesis that the author intends to investigate?
- Does the event describe well-established knowledge or new knowledge? New knowledge may correspond to direct observations, or an analyses made by the author based on experimental results

A text mining system that can distinguish between these different types of interpretations can clearly be useful to users. The ability to distinguish between new and well-established knowledge may be useful in applications, such as curating a database of known protein interactions.

In order to allow precise meta-knowledge to be recognized at the level of events, the annotation task described in this document will identify and assign different types of meta-knowledge to each individual event in a document.

1.2.1 Meta-Knowledge Examples

To make the ideas of meta-knowledge introduced above more concrete, let us consider 8 sample sentences, the majority of which contain 2 basic events:

1) A *positive regulation* event where *Y* is the AGENT, and the *expression* event described in 2) is the THEME
2) An event describing a *gene expression*, where *X* is the THEME

Note that, in most cases 1) is the primary event in the sentence, whilst 2) is the secondary event. It is normally the case that most of the meta-knowledge information expressed in the sentence will apply to the primary event. Often there is no information that allows a specific interpretation to be applied to a secondary event. This is not exclusively the case, although here we concentrate mainly on the interpretations of the primary events in the sentences.

The sample sentences are as follows:

(S3) ***We found*** *that Y activates the expression of X*

(S4) ***We examined*** *the effect of Y on expression of X*

(S5) *These results* ***suggest*** *that Y has* ***no*** *effect on expression of X*

(S6) *Y is* ***known*** *to increase expression of X*

(S7) *Addition of Y* ***slightly*** *increased the expression of X*

(S8) *These results* ***suggest*** *that Y* ***might*** *affect the expression of X*

(S9) ***Significant*** *expression of X was* ***observed***

(S10) ***Previous studies*** *have* ***shown*** *that Y activates the expression of X*

The trigger words for the events are underlined in each of the examples. The *expression* event, which occurs in all sentences, is always indicated by the nominalised verb *expression.* However, the positive regulation event is expressed in a number of different ways, namely using the verbs *activate*, *increase* and *affect*, or the nominalised verb *effect.* The positive regulation event occurs in all sentences, with the exception of (S9).

The emboldened words and phrases in the examples below help to show that the way in which the events should be interpreted can vary considerably. However, current text mining systems will normally treat the events extracted from all the above sentences in an identical way, thus missing important or even vital details about the event. Most of the emboldened words affect the interpretation of the positive regulation event, which is the main event in the sentence. However, in (S9) the interpretation of the expression event is altered.

In sentence (S3) above, the presence of the word *found* shows explicitly that the positive regulation event is backed by evidence, i.e. it is an experimental observation. The word *we* shows that is very likely that event was observed by the authors of the paper as part of the study being described, which would mean that it could be considered as "new" knowledge. No explicit information is specified for the secondary expression event, although we also consider this to be an observation.

The interpretation of the positive regulation event in (S10) is very similar to (S3). The presence of the word *shown* is again an explicit indication that the positive regulation event is an experimental outcome. However, the use of *Previous studies* at the start of the sentence indicates that these results were originally reported outside of the current paper, and hence the event should not be considered as not "new" knowledge. Once again, there is no explicit information regarding the secondary expression event, but again we would treat this as an observation

Sentence (S6) also contains events with similar interpretations to those in (S3) and (S10). However, the word *known* serves to indicate that the positive regulation event is a well established fact within the field. Whilst (S3) and (S6) can be seen as representing the same type knowledge at some level, in that they both report the event is a definite fact which is backed by evidence, the degree of the "reliability" of the events is subtly different, in that (S3) reports a new experimental outcome rather than well-established knowledge.

Whilst there are subtle differences in the interpretation of the positive regulation events in (S3), (S6) and (S10), they all have in common that the event is presented as without any expression of uncertainty. In this respect, the positive regulation event in (S4) is quite different. Here, the presence of the word "examined" serves to indicate that the positive regulation event is under examination, and so, at least at that point in the text, it is not possible to determine whether or not the event is true. Thus, it would be incorrect for a text mining system to present the positive regulation event in this context as a definite fact or an observation.

In (S8), there is yet a different interpretation of the positive regulation event. In using the word *might,* the author is indicating some amount of speculation towards the truth of the event. Furthermore, the use of the verb *suggests* denotes that the evidence for the author's tentative statement is based on some kind of analysis or inference drawn from results. Such evidence is, by its nature, less reliable than the direct evidence than was stated to be behind the positive regulation events in (S3), (S6) and (S10).

In sentence (S7), the word *slightly* provides explicit information about intensity of the positive regulation. In (S9), there is only one event, i.e. the expression event. Here, this event becomes the primary event in the sentence, even though its trigger in the nominalised verb *expression.* The intensity of the expression event is indicated, i.e., *significant*. The use of the word *observed* in this sentence shows that this expression event corresponds to an experimental observation.

From the above sentences, we can identify several important pieces of interpretative information which can be regularly deduced about events, according to the context in which they appear. These types of information modify the default interpretation (i.e. as positive, definite facts) of the events:

1) What kind of evidence is there for the event, e.g. has it been experimentally observed, inferred from experimental results, is a well established fact, or is it a hypothesis whose truth has yet to be determined?
2) How certain is the author about whether the event is true?
3) What is the source of the information contained within the event? Is it reported in the current paper or another paper?

The level of impact of each piece of contextual information varies from fairly subtle to fairly significant. However, even subtle information can be important, depending on the task being undertaken or the goals of the user. Therefore, we wish to perform annotations that will capture evidence in the text for all of the above types of information. The next section provides more details about the annotation scheme we have designed to allow the above types of information to be made explicit.

Clearly some examples will require wider contextual knowledge to make an informed decision for the new knowledge and hypothesis dimensions. To give the annotator the best chance of understanding the context of a piece, we have include the entire abstract or paragraph that an event came from in the annotation task.

2 The Annotation Scheme

Based on the types of meta-knowledge highlighted in the previous section, which appear to be most pertinent to the interpretation of bio-events, we have defined a scheme to annotate these within biomedical texts.

At the heart of scheme are 2 key meta-knowledge *dimensions*, which are called *Hypothesis and New Knowledge* (Figure 2). The other boxes in figure 2 show the types of information that have typically previously been annotated for events in biomedical texts. Each of the meta-knowledge dimensions, which are described in detail in the following subsections, corresponds to a particular type of meta-knowledge. The annotation task is as follows: For each event, determining an appropriate value (from a fixed set) for each dimension, based on evidence from the context in which the event occurs (e.g., the sentence in which the event is described, or previous sentences). The type of evidence that is present can vary. Most often, the presence of particular word or phrase in the same sentence is used as the evidence. In other cases, the evidence constitutes another feature of the sentence, or even the position of the sentence within the abstract.

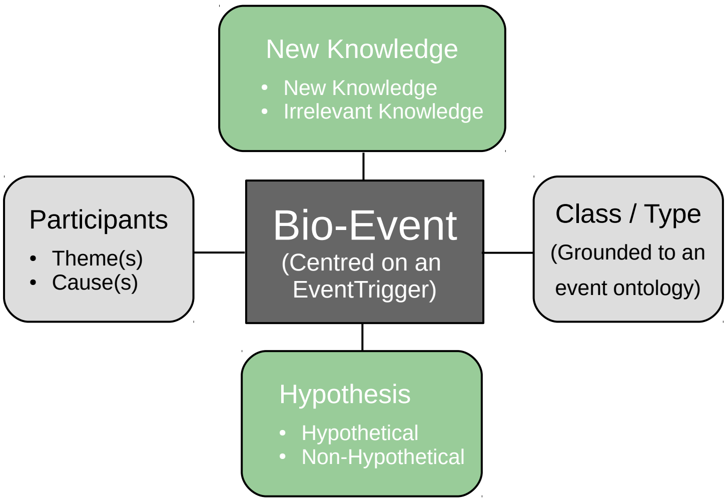

Figure 2. The Meta-Knowledge Dimensions

The purpose of the annotation, then, is to discover the different ways in which each value of each dimension can manifest itself as evidence in the text. When we have annotated a large enough set of documents, we can train a system to learn patterns based on these annotations. The trained system will then be able to predict the values of the annotation dimensions for previously unseen events. In the following sections, we provide detailed information regarding the 5 individual meta-knowledge dimensions. A brief description of each dimension is followed by an enumeration of its possible values, together with some examples. In all of the examples, the word(s) on which the event is centered (i.e. the trigger word/phrase) are shown using *underlined italics*, whilst the explicit “clue” words which provide evidence for the assignment of a particular value to a dimension are shown using **bold face.**

2.1 New Knowledge

New Knowledge indicates that the author intends the reader to interpret the statement as a new piece of information. Any directly reported information from the background literature should be classified as irrelevant knowledge. Irrelevant knowledge is used as an umbrella term for any knowledge that the author does not intend the reader to digest as the main discourse of the paper – it can be considered therefore as irrelevant to their ultimate findings. During the annotation task, an event should be classified as 'irrelevant knowledge' if there is some explicit indication from the context that it is either from the background literature, or otherwise not introduced directly as a part of the paper. There is some degree of subjectivity to this classification and we encourage the annotators to employ their own discretion to decide whether a statement is intended by the author as a new piece of knowledge for the reader.

2.1.1 New Knowledge

- Evidence
  1. Novel information being presented (typically may have knowledge type of analysis or observation)
- Example Sentences

(S86) It was found that lipopolysaccharide induced strongly both c-fos and c-jun expression as well as AP1 formation. *(in discussion / results section)*

(S87) We report here that the second alteration, at threonine 78, also plays an important, although more indirect, role.

2.1.2 Irrelevant Knowledge

- Evidence

1. May come from the background literature
2. Knowledge which is current, but not directly part of results, etc.

- Example Sentences

(S88) In addition, pretreatment of the cells with the proteasome inhibitor N-Ac-Leu-Leu-norleucinal inhibits this ligand-induced degradation and, in agreement with previous studies, stabilizes a hyperphosphorylated form of the human I kappa B alpha protein.

(S89) Since previous studies have demonstrated that the c-jun gene is *autoinduced* by Jun/AP-1, we also studied transcription of c-jun promoter (positions -132/+170)-reporter gene constructs with and without a mutated AP-1 element.

- Discussion of Examples

New knowledge is clearly intrinsically linked with the source of the knowledge. It will always be the case that if an event is from the background literature then we will have 'irrelevant knowledge'. If the source happens to be current then we must consider if the event is introducing knowledge as a direct result of the findings in the paper at hand. The annotator should assume that the knowledge is part of the findings from the paper, unless there is a clear indication otherwise as in (S88), where the source is clearly current as the authors are talking about their own work – but the information contained in the snippet is not a result of the author's work.

2.2 Hypothesis

Hypothetical statements indicate something that the author is trying to discover about the subject of the paper, i.e. the question that the author is trying to ask. An event is considered hypothetical when the context indicates that the author is explicitly discussing their research hypotheses, or generally talking about what they wish to learn from the experiment that they intend to conduct. Statements of what the author has discovered, whether previously or in the current work, should not be considered hypotheses – unless they are explicitly presented in a hypothetical manner (e.g., "previously we hypothesized that…"). One paper may contain multiple hypotheses, if the author is trying to learn about several things at once. Events in titles often convey hypotheses, although these may be stated without clear clues. The main event in a title should be considered for marking as a hypothesis.

2.2.1 Hypothetical

- Evidence

1. The author is making a hypothesis about the event in question
2. The author is describing what they were trying to discover
3. Typical clues include: *we hypothesise, we investigate, we think…*

- Example Sentences

(S90) We therefore hypothesized that the release of KRas from the PM results from the Ca2+-dependent binding of a cytoplasmic protein to the COOH-terminal tail of KRas, and subsequent destabilization of KRas interactions with the PM

(S91) This prompted us to investigate whether CaM mediates KRas and Rap1a translocation in neurons by interacting with their membrane anchors.

2.2.2 Non-Hypothetical

- <u>Evidence</u>
  1. No clear sense of a hypothesis
  2. Typically, a statement of fact, method or experimental observation
- Example Sentences

  (S92) Oxidants such as hydrogen peroxide are **known** to <u>*activate*</u> certain transcription factors such as nuclear transcription factor kappa beta.

- <u>Discussion of Examples</u>

  The annotator should consider an example to be hypothetical only if there is specific evidence that the author is drawing upon their own knowledge or analysis to make a statement about an event. We see this in S90 where that author is using the phrase 'we hypothesised' – this indicates that they are explicitly talking about a hypothesis they intended to make. We also see this in S91, where the author uses the phrase 'this prompted us to investigate'. Here they are discussing what they intend to find and so again it is classed as a hypothesis. If no specific evidence for hypothesis exists (as in S92) then the sentence should be classified as non-hypothetical.

3 Examples

Having examined in the different annotation dimensions of the scheme in some detail, we now re-examine the hypothetical sentences first introduced in section 1.2.1, and discuss the correct categories to assign to them for each meta-knowledge dimension.

(S3) *We **found** that Y activates the expression of X*

Event 1: *activates*

Hypothesis: Non-Hypothetical

New Knowledge: New Knowledge

Event 2: *expression*

Hypothesis: Non-Hypothetical

New Knowledge: Irrelevant Knowledge

(S4) *We **examine** the effect of Y on expression of X*

Event 1: *activates*

Hypothesis: Hypothetical

New Knowledge: Irrelevant Knowledge

Event 2: *expression*

Hypothesis: Non-Hypothetical

New Knowledge: Irrelevant Knowledge

(S5) *These results **suggest** that Y has **no** effect on expression of X*

Event 1: *effect*

Hypothesis: Non-Hypothetical

New Knowledge: New Knowledge

Event 2: *expression*

Hypothesis: Non-Hypothetical

New Knowledge: Irrelevant Knowledge

(S6) *Y is **known** to increase expression of X*

Event 1: *increase*

Hypothesis: Non-Hypothetical

New Knowledge: Irrelevant Knowledge

Event 2: *expression*

Hypothesis: Non-Hypothetical

New Knowledge: Irrelevant Knowledge

(S7) *Addition of Y* ***slightly*** *increased the expression of X*

Event 1: *increased*

Hypothesis: Non-Hypothetical

New Knowledge: New Knowledge

Event 2: *expression*

Hypothesis: Non-Hypothetical

New Knowledge: Irrelevant Knowledge

(S8) *These results* ***suggest*** *that Y* ***might*** *affect the expression of X*

Event 1: *affect*

Hypothesis: Non-Hypothetical

New Knowledge: New Knowledge

Event 2: *expression*

Hypothesis: Non-Hypothetical

New Knowledge: Irrelevant Knowledge

(S9) ***Significant*** *expression of X was* ***observed***

Event 1: *expression*

Hypothesis: Non-Hypothetical

New Knowledge: New Knowledge

(S10) ***Previous studies*** *have* ***shown*** *that Y activates the expression of X*

Event 1: *activates*

Hypothesis: Non-Hypothetical

New Knowledge: Irrelevant Knowledge

Event 2: *expression*

Hypothesis: Non-Hypothetical

New Knowledge: Irrelevant Knowledge

4 Annotation Task

In the previous section, the annotation was annotated from a slightly abstract point of view, in that detailed information was not given regarding the events on top of which the meta-knowledge will be performed, or about the steps involved in the annotation task. This section addresses these aspects of the task in more detail.

4.1 What Annotation is Already There?

The annotator will be presented with sentences over which annotations of the following types have been automatically generated: (1) named entities, and (2) events. A sample sentence is depicted in Figure 4 below.

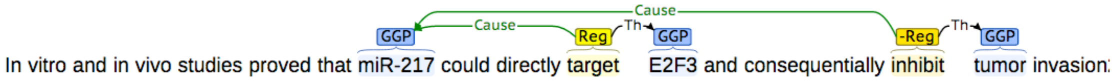

Figure 4 – A sample sentence annotated with named entities and events.

Named entities appear as text spans with labels that are indicative of what type of biomolecular entities they are, e.g., gene or gene product (GGP), simple chemical, protein complex, cellular component. Marked up as well are biomolecular events, structured annotations which capture typed, *n*-ary relationships between entities. Figure 4, for instance, shows two events: the regulation (Reg) event anchored to the trigger word *target* involving *miR-217* and *E2F3*, and the negative regulation (-Reg) event having *inhibit* as its trigger and *miR-217* and *tumor* as its participants.

4.2 What does the annotation task involve?

For this task, the annotator should focus only on providing values of meta-knowledge attributes for each event. Named entity and event annotations are not to be modified, even if they seem incorrect.

The following meta-knowledge attributes of each event need to be assigned values by the annotator: Hypothesis and New Knowledge.

4.3 Which events should I annotate with meta-knowledge

An event is a relationship between two or more entities, where an entity may be a single term or another event. Principally annotators should try to recognise the main event in a sentence and add meta-knowledge to this. For example, in S3 we saw that the main event was "Y **activates** expression of X", i.e. an activation event between 'Y' and 'expression of X'. We also see that there is a secondary event here "**expression** of X", i.e. that X is being expressed. In this sentence, the authors are communicating that they have found out something about the primary activation event and so we annotate this primary event as new knowledge. The authors are not however communicating that they have found out something new about the secondary expression event. This may well refer to something that is already well known and should therefore not be considered new knowledge. Annotators may wish to keep track of the primary or secondary events using the notes feature in the annotation tool.

4.4 Sequential Events

In many cases an author may write a list of events, as in the following example:

(S11) *We **found** that Y **actives** the **expression** of A, B and C.*

In S11, there are 6 events to consider, as follows:

- **E1:** Expression of A
- **E2:** Expression of B
- **E3:** Expression of C
- **E4:** Activation of **E1**
- **E4:** Activation of **E2**
- **E4:** Activation of **E3**

Given the clue 'found' we are aware that there is some new knowledge present in this sentence. As previously, we identify the primary event as **activation** and the secondary event as **expression**. It is important that the annotator remembers to identify all 3 activation events and mark these up with the relevant new knowledge. The three expression events should not be annotated as new knowledge.

5 Annotation Environment

For this task, the annotator will use the brat rapid annotation tool (brat) to provide his/her annotations. This tool runs as a web application and does not require any specialised software apart from a standard web browser, e.g., Google Chrome, Firefox or Safari.

5.1.1 User login

brat requires a user to be logged in to the system in order to create or modify annotations (Figure 5). We shall provide annotators with their login details together with the URL to the specific data set they will be working on.

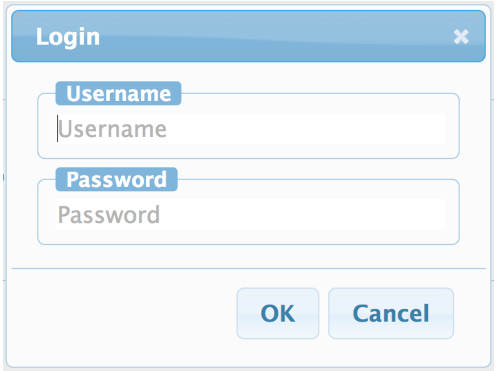

Figure 5 – An annotator needs to provide his/her login details in brat's login window.

5.1.2 Document navigation

An annotator can navigate through the documents for annotation using brat's document browser. By default, the documents are listed by alphabetical order of filenames, but they can also be sorted by modification time or number of entities/events contained.

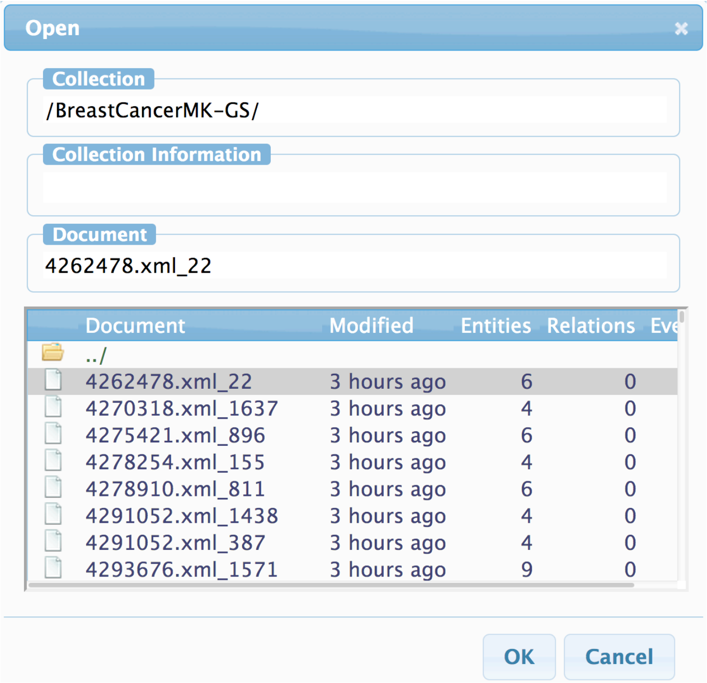

Figure 6 – brat's document browser.

Once a document has been displayed, the annotator can also use the arrow buttons on the upper left-hand side of the screen (Figure 7) to navigate to other documents in the corpus.

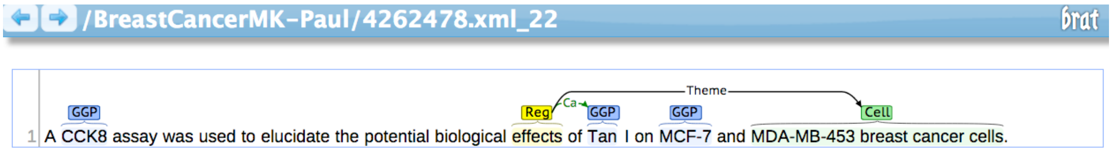

Figure 7 – A document displayed in brat.

5.1.3 Meta-knowledge annotation

To assign values to any of the meta-knowledge attributes, one has to double-click on the event trigger annotation to bring up the window displaying the event's attributes (see bottom panel of the window in Figure 8). Annotators should refrain from modifying the values of other event attributes, e.g., Event type.

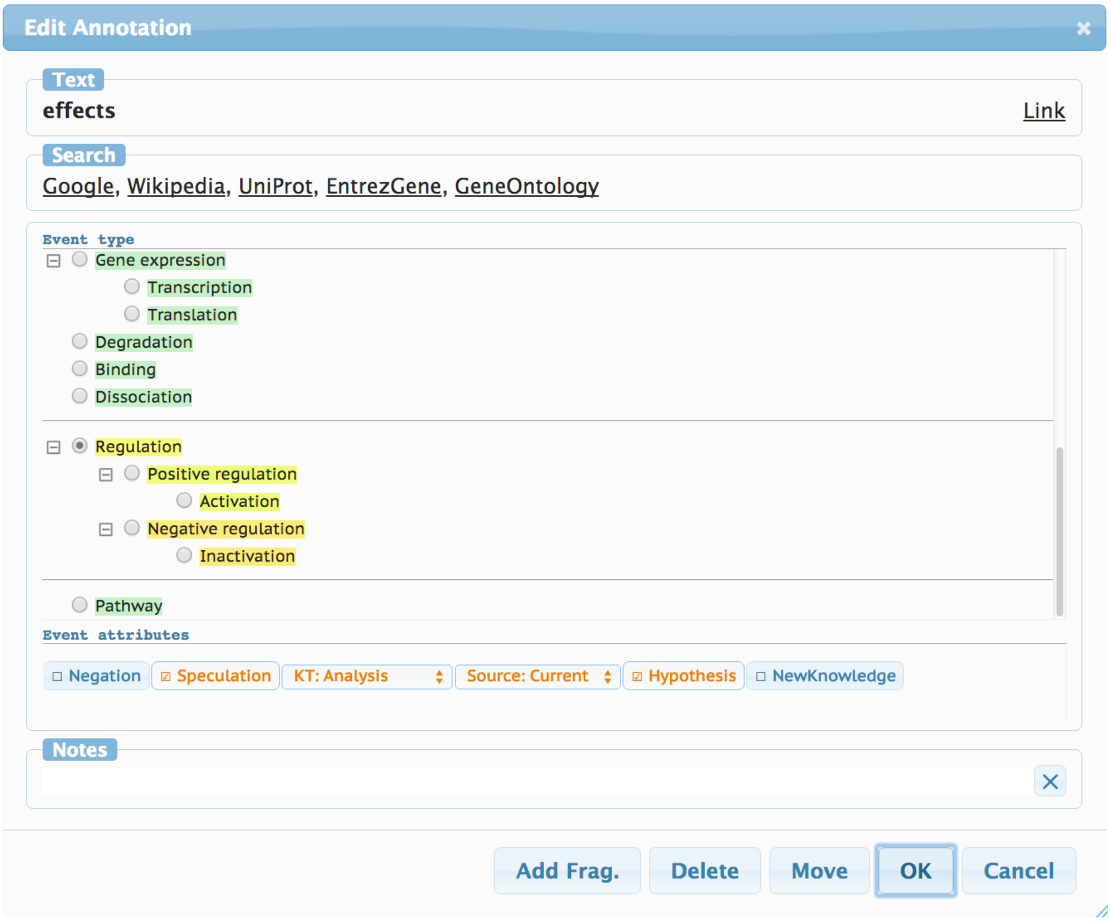

Figure 8 – Window for editing event meta-knowledge attributes (bottom panel).

Meta-knowledge attributes which are boolean-valued, e.g., Negation, Speculation, Hypothesis, New Knowledge and Background appear as tick boxes. To indicate that an event is negated, speculated or that it corresponds to a hypothesis, new knowledge, or background information, the annotator should tick the relevant box. For the other meta-knowledge attributes, e.g., Knowledge Type and Source, possible values appear in the form of drop-down lists from which the annotator should select the value he/she wishes to assign.

5.1.4 Providing points for discussion

If the annotator wishes to raise any question or point for discussion, he/she can use the Notes field at the bottom of the Edit Annotation windows (Figure 8) to type in any comments
