# Supplementary material for: Identification of research hypotheses and new knowledge from scientific literature
Source: BMC Med Inform Decis Mak. 2018 Jun 25;18:46. doi: 10.1186/s12911-018-0639-1 (PMC6019216; doi:10.1186/s12911-018-0639-1)
Supplement: Supplementary file 2 — A table providing an in depth description of each feature. (PDF 32 kb) [file 12911_2018_639_MOESM2_ESM.pdf]

Category	Index	Name	Description	Example sentence	Example value
Sentence	1	Length in words	The total length of the sentence in terms of the number of tokens. token boundaries were determined by parsing the sentence using the Enju parser.	*We examined the possibility of establishing new cell lines.*	9
	2	Length in characters	The total length of the sentence in terms of the number of characters. All non-whitespace characters are included.	*We tested the hypothesis that oral beclomethasone dipropionate (BDP) would control gastrointestinal graft-versus-host disease.*	113
	3	Mean number of characters per word	The mean is calculated as the total number of characters (feature 2) divided by the total number of words (feature 1)	*CTCF is a transcriptional repressor of the c-myc gene.*	5.11
	4	Median number of characters per word	The median value is calculated by looking at an ordered list of the character values for each word and selecting the middle value. if an even number of words is present we take the aevrage between the middle two values.	*We show that Oral BDP prevents relapses of gastrointestinal GVHD*	4
	5	Noun-to-verb ratio	The number of nouns in the sentence divided by the number of verbs	*To investigate the role of Interleukin-17 (IL-17) in the pathogenesis of psoriasis*	4
	6	Noun-to-adjective ratio	The number of nouns in the sentence divided by the number of adjectives	*IL-17 is a critical factor in the pathogenesis of psoriasis and other inflammatory diseases.*	2.5
	7	Noun-to-adverb ratio	The number of nouns in the sentence divided by the number of adverbs	*these findings strongly support our conclusion.*	2
	8	Verb-to-adjective ratio	The number of verbs in the sentence divided by the number of adjectives	*IL-17 is a critical factor in the pathogenesis of psoriasis and other inflammatory diseases.*	0.5
	9	Adjective-to-adverb ratio	The number of adjectives in the sentence divided by the number of adverbs	*these findings strongly support our conclusion that IL-17 is a critical factor in the pathogenesis of psoriasis.*	1
Event	10	Participant is an event	True if any particpant of the event in question is an event itself. In the example the main event is *activates* and its theme is the secondary event *expression*. We are generating the feature for *activates*.	*y activates the expression of x*	True
	11	Event is a participant	True if the event is a participant in another event. In the example the main event is *activates* and its theme is the secondary event *expression*. We are generating the feature for *expression*.	*y activates the expression of x*	True
	12	Number of themes	The total number of themes associated with an event. In the example the event is centred around activated. y1, y2 and y3 are each separate themes	*We found that x activated y1, y2 and y3*	3
	13	Number of causes	The total number of causes associated with an event. In the example the event is centred around activated. x1, x2 and x3 are each separate causes	*We found that x1, x2 and x3 activated y*	3
	14	POS tag of first theme	The part of speech of the first participant (typically this will be a noun, although might be a verb in some cases)	*The narL gene product activates the nitrate reductase operon*	noun (gene product)

	15	POS tag of first cause	The part of speech of the first participant (typically this will be a noun, although might be a verb in some cases)	*The narL gene product activates the nitrate reductase operon*	noun (reductase operon)
	16	Any theme is an event	True if any of the themes is an event	*We found that x activated y1, y2 and expression of y3*	true
	17	Any cause is an event	True if any of the causes is an event	*We found that x1, x2 and x3 activated y*	true
	18	Part-of-Speech tag of theme dependency	Uses a dependency parser to identify syntactic relations between words. This feature gives the Part-of-Speech tag of the dependency of the theme. The Part-of-Speech defines the role of the word in the sentence (e.g., noun, verb, adjective, etc.). The dependency parser tells us which words are syntactically associated. In the example, there is a dependency between *activates* and *operon*, which shows that operon is the object of the verb *activates*. The dependency of the theme will usually be the trigger of the event.	*The narL gene product activates the nitrate reductase operon*	verb
	19	Part-of-Speech tag of cause dependency	This feature gives the Part-of-Speech tag of the dependency of the cause. The dependency of the cause will usually be the trigger of the event. In this case there is a dependency between *product* and *activates*, indicating that *product* is the subject of the verb activates.	*The narL gene product activates the nitrate reductase operon*	verb
Lexical	20	Contains a clue	True if any clues from a precompiled list were found in the sentence that contained this list. In the example *found* is a clue for new knowledge.	*We found that Y activates the expression of X*	True
	21_n	Clue N present	A set of N features, where N is the size of the clue list. Each feature indicates whether one specific clue was available. In the example *significant* and *observed* are both clues, and as such would correspond to separate features	*Significant expression of X was observed*	True
	22	Number of matched clues	The total number of clues that were found in the sentence	*Significant expression of X was observed*	2
	23	Distance between nearest clue and trigger	The number of tokens between the event trigger and the nearest clue in the sentence. Set to the furthest sentence boundary if no clue is present. In the example there are 2 tokens between *found* and *activates.*	*We found that Y activates the expression of X*	2
	24	Surface form of clue	The raw form of the nearest clue to the trigger	*We found that Y activates the expression of X*	found
	25	POS tag of clue	The part of speech of the nearest clue to the trigger	*We found that Y activates the expression of X*	verb
	26	Position relative to trigger	Whether the nearest clue was found before or after the event trigger	*We found that Y activates the expression of X*	before
	27	Clue in auxiliary form	true if the nearest clue was in auxillary form (qualified with 'have' 'be', etc.). In the example, this feature is true as the clue *observed* is qualified by *will be.*	*expression of X will be observed*	true
	28	Trigger contains a clue	True if the event trigger itself contains a clue	*We found that Y activates the expression of X*	False

	29	Tense of clue	Past, present or future - indicates temporal information about the metaknowledge associated with the event	*Addition of Y slightly increased the expression of X*	past
	30	Aspect of clue	indicates whether the nearest clue is expressed as an action in an ongoing state.	*y is activating x*	true
	31	Voice of clue	indicates whether the nearest clue is written in the active or passive voice. Passive voice sentences are qualified with the verb 'to be'	*significant expression of x was observed*	true
	32_n	Whether clue usually occurs in the context of each knowledge type	A separate feature for each knowledge type (Observation, Investigation, Analysis, Fact, Method, Other), indicating whether a clue pertaining to each of these knowledge types was discovered.	*Significant expression of X was discovered*	Analysis: True
Constituency	33	S-commands relation between clue and event trigger	True if the S-commands relation holds in the constituency parse tree between the clue and the event trigger. Effectively testing if the clue is in the same sentence as the trigger	*Significant expression of X was observed*	True
	34	VP-commands relation between clue and event trigger	True if the VP-commands relation holds in the constituency parse tree between the clue and the event trigger. Effectively testing if the clue is in the same verb phrase as the trigger	*We observed expression of X*	True
	35	NP-commands relation between clue and event trigger	True if the NP-commands relation holds in the constituency parse tree between the clue and the event trigger. Effectively testing if the clue is in the same noun phrase as the trigger	*significant expression of X*	True
	36	Relationships between clue and any event participant	true if any of the above relations (33, 34 or 35 is true)	*We observed significant expression of X*	True
	37	Whether scope of clue is in the same scope as the trigger	Indicates whether the scope of the clue (i.e. the part of the text annotated as the clue) intersects with the scope of the event (i.e. the part of the text annotated as the event). The scope of the event is defined as all the text enclosed by the trigger and participants. In the example, the clue *slightly* occurs within the event which begins at *Addition* and ends at *X*.	*Addition of Y slightly increased the expression of X*	True
Dependency	38	Direct dependency between clue and trigger	True if there is a direct dependency between the clue and the event trigger. In the example there is a direct dependency between *observed* and *expression*.	*we observed significant expression of X*	True
	39	Direct dependency between clue and event participant	True if there is a direct dependency between the clue and any event participant. In the example *observed* is a clue and *expression* is a participant of the event *increased*.	*Y increased the observed expression of X*	True
	40	One-hop dependencies between clue and trigger	As above, but with a 'one hop' dependency. i.e., the clue has a dependency, which in turn has a dependency, which is the trigger. In the example, the dependency path goes *observed* → *existed* → *expression*	*We observed that there existed significant expression of X*	True

	41	One-hop dependencies between clue and event participant	As above, but with a 'one hop' dependency. i.e., the participant has a dependency, which in turn has a dependency, which is the participant. In the example, the dependency path goes *shown* → *activates* → *X*	*we have shown that Y activates X*	True
	42	Two-hop dependencies between clue and trigger	As for one-hop, but for two hops, instead of one. In the example, the dependency path is *Significantly* → *shown* → *existed* → *expression.* Where *significantly* is the clue and *expression* is the event trigger.	*Significantly, we have shown that there existed a strong expression of X*	True
	43	Two-hop dependencies between clue and event participant	As for one-hop, but for two hops, instead of one. In the example, the dependency path is *observed* → *activation* → *of* → *X.*	*we observed activation of X*	True
Parse Tree	44	Distance between theme and furthest leaf node	the number of nodes in the parse tree between the first theme and the deepest leaf node beneath it. Parse tree depth is calculated as the number of nodes between the current node and the root node. distance is calculated as the difference between parse tree depth. See Figure 1 for the parse tree. In the example *expression* is the theme and *X* is the deepest leaf node beneath it.	*Addition of Y slightly increased the expression of X*	2
	45	Distance between cause and furthest leaf node	the number of nodes in the parse tree between the first cause and the deepest leaf node beneath it. Parse tree depth is calculated as the number of nodes between the current node and the root node. distance is calculated as the difference between parse tree depth See Figure 1 for the parse tree. In the example *Addition* is the cause and *Y* is the deepest leaf node beneath it.	*Addition of Y slightly increased the expression of X*	3
	46	Distance between theme and root node	the number of nodes in the parse tree between the first theme and the root node at the top of the tree. See Figure 1 for the parse tree. In the example *expression* is the theme.	*Addition of Y slightly increased the expression of X*	5
	47	Distance between cause and root node	the number of nodes in the parse tree between the first cause and the root node at the top of the tree. See Figure 1 for the parse tree. In the example *Addition* is the cause.	*Addition of Y slightly increased the expression of X*	8

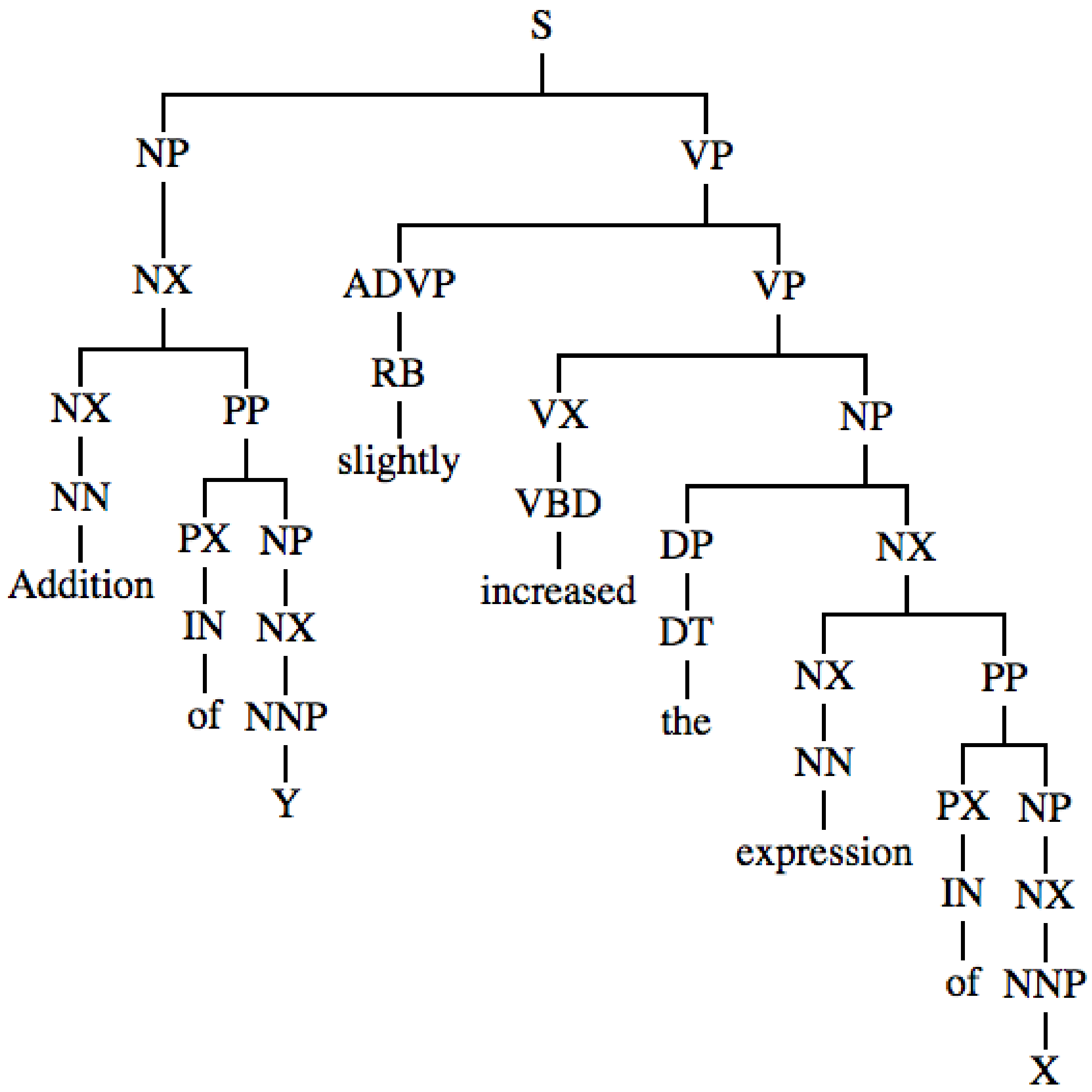

Figure 1: The parse tree for the sentence "Addition of X slightly increased the expression of X" as observed in features 44–47.
